# Scientific justifications for the political decision-making on environmental remediation carried out after the Fukushima nuclear accident

**DOI:** 10.1016/j.heliyon.2021.e06588

**Published:** 2021-03-29

**Authors:** Maria R.H. Takeuchi, Tatsuya Hasegawa, Susie M.L. Hardie, Linda E. McKinley, Gian Powell B. Marquez, Keiichi N. Ishihara

**Affiliations:** a Graduate School of Energy Science, Kyoto University, Yoshidahonmachi, Sakyo-ku, Kyoto 606-8501, Japan; b Department of Aerospace Engineering, Graduate School of Engineering, Nagoya University, Furo-cho, Chikusa-ku, Nagoya 464-8603, Japan; c McKinley Consulting, Oberer Rainweg 15, Frick, 5070, Switzerland; d College of Global Liberal Arts, Ritsumeikan University, Ibaraki Osaka 567-8570, Japan

**Keywords:** Environmental remediation, Nuclear power, Nuclear accidents, Waste management, Decision-making, Leadership

## Abstract

The Japanese government decided to implement environmental remediation after the Fukushima Daiichi Nuclear Power Plant (termed “1F” in Japan) accident on 11th March 2011. As the initial additional annual dose target was set to be 1 mSv or less as a long-term goal, we examined the decision-making process undertaken by the then leaders, particularly the Minister of the Ministry of the Environment (MOE) who was responsible for the final decision. We found that technically based assessment of dose targets, health effects and risk-based approaches justified by scientific experts were not communicated to the then Minister and officials of the MOE before the remediation strategy was decided. We defined how such a decision was made based on leadership theories such as the Role Theory and the Cognitive Resources Theory. Academic leaders could have examined the Windscale accident (UK, 1957), which could be considered as the closest analogue (at least in terms of radionuclide releases) to the 1F accident. Environmental remediation could have been planned and implemented more effectively whilst still maintaining the highest possible safety standards and balancing the environmental and economic burden. Appropriate scientific input should have been provided by academic leaders to political and administrative leaders and such scientific justification should have been disclosed to the general public (especially the residents of Fukushima Prefecture) so that the general public could have developed greater trust in their leaders and have more readily accepted the decisions made.

## 1 Introduction

Following the Great East Japan Earthquake and Tsunami on 11th March 2011 and the subsequent reactor meltdowns at the Fukushima Daiichi (termed “1F” in Japan) Nuclear Power Plant (NPP), the Japanese government was directly responsible for making key decisions in terms of both immediate responses and long-term recovery of the devastated coastal area on the northeast of the main island (Honshu) of Japan [1, 2] (Table 1). After stabilization of the 1F site, the Japanese government believed environmental remediation was crucial and decided to implement a program to classify and remediate the evacuated areas. They conducted clean-up work to remove radioactive materials in Special Decontamination Areas (SDA) where the fallout of radionuclides was higher under the direct control of the Japanese government. The SDA included the Evacuation Zone (20 km from the Nuclear Power Plant) and the Planned Evacuation Zone (annual cumulative dose of >20 mSv). Municipalities in the Intensive Contamination Survey Areas (ICSA) also conducted such clean-up based on radiation surveys with technical and financial support from the Japanese government. The areas, where an additional exposure dose (over and above natural background radiation) of over 1 mSv/year was observed, were designated as ICSA [8, 9]. The Japanese government invested huge financial and human resources in order to enable the fastest possible return of evacuees [1].

**Table 1:** The chronological order of events of the Fukushima Daiichi Nuclear Power Plant accident on March 11, 2011.

Date	Events
March 11, 2011	Earthquake and tsunami happened which caused loss of all electricity in Unit 1, 2 and 4 reactors and blackout in Unit 3. Unit 1 reactor started to have core damage [1].
March 12, 2011	Hydrogen explosion happened at the reactor building of Unit 1 [1].
March 13, 2011	Unit 3 reactor started to have core damage [1].
March 14, 2011	Hydrogen explosion happened at the reactor building of Unit 3. Unit 2 reactor started to have core damage [1].
March 15, 2011	Massive radioactive material discharge from Unit 2 due to damage suppression chamber. Hydrogen explosion happened at the reactor building of Unit 4. Residents living between 20 km and 30 km from the plant were ordered to shelter-in-place [1].
March 25, 2011	Public call for voluntary evacuation was announced while shelter-in-place was in effect [1].
April 22, 2011	Planned evacuation was ordered by the government [1].
July 19, 2011	The Nuclear Safety Commission (NSC) stated the basic view that a lower reference level should be selected from the additional dose range of 1–20 mSv/year and set the long-term goal of an additional dose of no more than 1 mSv/year for residents which is based on the ICRP Publication 103 [3,4].
August 26, 2011	The Nuclear Emergency Response Headquarters decided to base their fundamental policy on urgent implementation of environmental remediation from the ICRP Publication 103 and basic view of NSC [5].
August 30, 2011	Passing of legislation, “Act on Special Measures concerning the Handling of Radioactive Pollution” (Act No. 110 of 2011), by Diet members [6].
October 2, 2011	The Minister of the Ministry of the Environment (MOE) announced to the Governor of Fukushima prefecture that they would aim to reduce additional annual doses (over and above natural background radiation) to 1 mSv or less as a long-term goal [7].
December 22, 2011	The MOE published the Decontamination Guidelines to correspond to the implementation of the Act No. 110 of 2011 [8].
January 1, 2012	The Act No. 110 of 2011 came into force [8].

Immediately after the 1F accident, comparisons were made with the Chernobyl accident and the Chernobyl exclusion zone (CEZ) by the media, even though many technical experts were fully aware of the differences between these two accidents as previously pointed out by Hardie and McKinley [10]. On the basis of the scientific facts, the fallout from the 1F accident was recognized to be more akin to either the fallout from Windscale or the distant Chernobyl fallout deposited in Fenno-Scandinavia and parts of the United Kingdom than that in the Chernobyl exclusion zone [10] (Table 2).

**Table 2:** The nuclear reactor accidents of Chernobyl, Windscale, and Fukushima.

Chernobyl (1986; Ukraine former USSR) [10]	• Criticality excursion during tests. • Explosive release of core contents. • Long-term releases during/after responses to control fire/criticality.
Windscale (1957; Cumbria, UK) [10, 11, 12]	• Core fire during secret production of Polonium. • Extensive releases of volatile components and water used to fight fire.
Fukushima (2011; Fukushima, Japan) [1, 10]	• Core melt due to heat decay and fuel pond damage after loss of power following tsunami.

The accident at the Chernobyl NPP reactor number 4 was caused by overheating during a safety test which led to a vapor explosion in the reactor core and subsequent fire. This resulted in the explosive dispersion of a large quantities of both volatile and non-volatile radioactive materials in the form of gases, fine aerosol mists and pieces of reactor core into the surrounding environment. The 1F accident, on the other hand, was triggered by hydrogen explosions in all three of the on-line reactors at the 1F plant. Just after the earthquake and subsequent tsunami, all three online reactors (units 1, 2 and 3) automatically shut down (scrammed) as they were designed to do and many of the very short-half-life radionuclides therefore decayed before core meltdown. The World Nuclear Association summarized the radioactive release from the Fukushima Daiichi reactors as “Major fuel melting occurred early on in all three units, though the fuel remains essentially contained except for some volatile fission products vented early on… [2].”

Radioactive gases such as the noble gases and radioiodine and lower boiling point radioactive metals such as cesium were dispersed into the environment after the accident. Otosaka *et al.* [13] concluded that most cesium was dispersed into the environment within one month after the accident. The focus of radiological assessment was mostly on ^131^I (half-life 8 days), ^137^Cs (half-life approximately 30 years) and ^134^Cs (half-life 2.1 years) due to environmental or human concentration mechanisms [10]. Miyahara and Ohara [14] concluded that radionuclides released from 1F were about 10% of the release during the Chernobyl accident, with the exception of the noble gases, and 80% of the radionuclides released from 1F went into the sea (including via aerial deposition).

The initial health focus was on radioiodine, particularly ^131^I due to its short half-life and potential to concentrate in both foodstuffs and the human thyroid. However, ^131^I decayed to insignificance within 3 months [10], after which the measured doses were mainly due to radioactive isotopes of cesium (specifically ^134^Cs, due to its much shorter 2-year half-life) according to field investigations carried out in June 2011 [15]. Koizumi *et al.* [16] pointed out that the rapid decay of the most hazardous short-lived isotopes and “natural cleaning” of longer-lived contaminants (e.g. cesium-137) reduced radiological health hazards considerably. It should be noted that although (radio-)cesium is strongly adsorbed by clay minerals and is not easily removed due to the strong affinity of clays for cesium [17], these clays can be mobilized in the environment, e.g. as suspended sediments in rivers.

Although it has been argued that there were many opportunities for the Japanese government, the regulators and the Tokyo Electric Power Co., Inc. (TEPCO) to strengthen measures that could have prevented the accident prior to 11^th^ March [1], this paper focuses on the situation that the government was then faced with: a globally unprecedented disaster in the absence of any kind of guidelines on which to base responses. Here, consideration is confined to actions off-site – which were decoupled from decision-making associated with management of the evolving situation at the 1F nuclear power plants.

In May 2011, the headquarters of the Fukushima Partnership Operations of JAEA were established in May 2011 to coordinate environmental remediation within Fukushima Prefecture and provide technical support to the MOE. Two model projects for environmental remediation were started outside the evacuated zone, in Minamisoma City and Date City (but still within Fukushima Prefecture) in August 2011. The guidelines subsequently produced for further clean-up were developed from the experience gained and lessons learned during the execution of these model projects [18].

**Figure 1:**
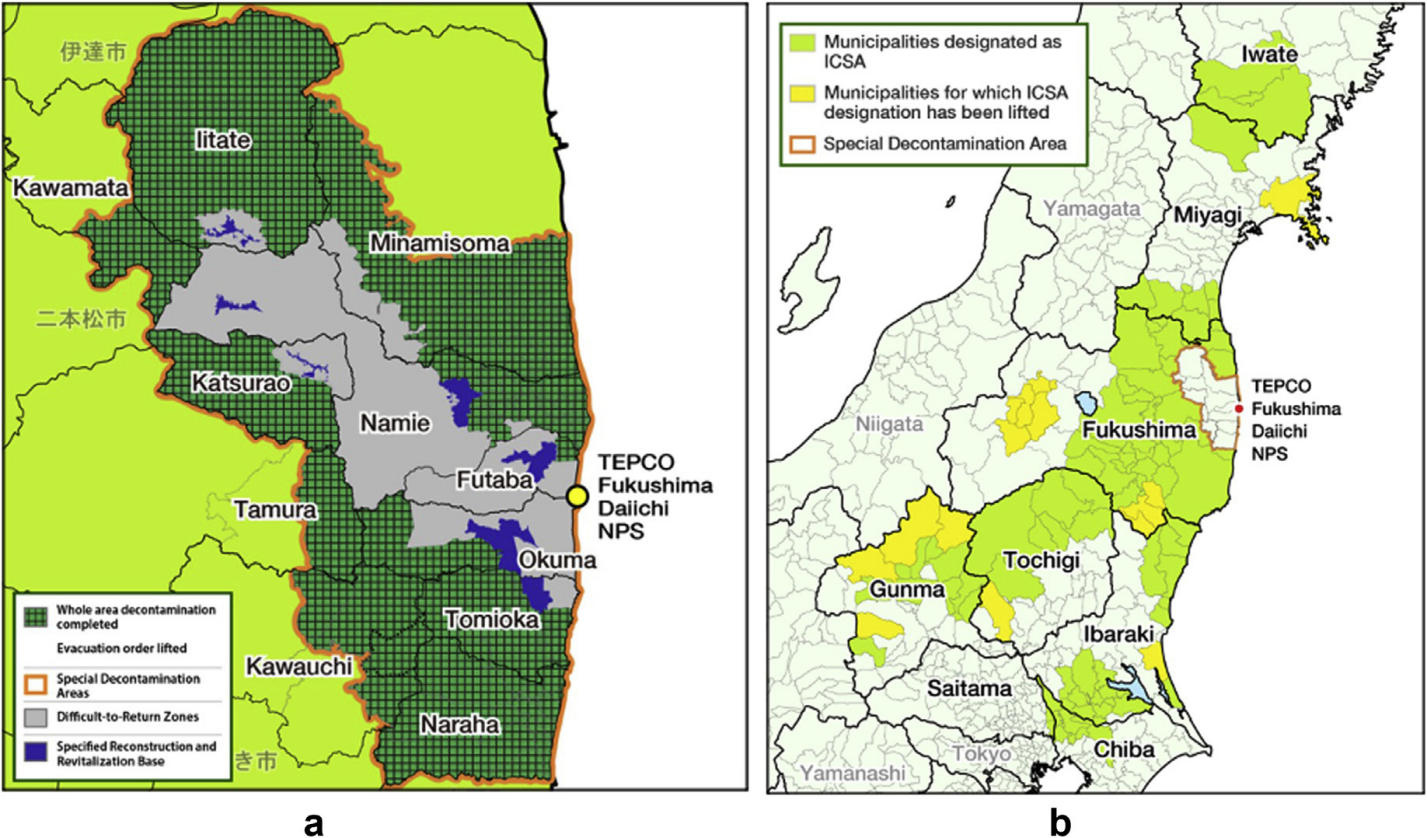
The areas in northeastern Japan showing the progress of decontamination **(a)** among eleven municipalities under the designation of the Special Decontamination Areas (SDA) and **(b)** within the Intensive Contamination Survey Areas (ICSA) as of March 19, 2018. Maps reproduced with permission of the Ministry of the Environment (MOE), Japan [21]. TEPCO - Tokyo Electric Power Company; NPS – Nuclear Power Station.

In order to develop and test tools and methodologies for decontamination, a further eleven demonstration model projects commenced in September 2011, this time within the evacuated zone. These demonstration model projects were carried out by three consortia, including major civil engineering contractors [19], after which region-wide remediation followed. Eleven Fukushima Prefecture municipalities in the SDA (basically the “Planned Evacuation Zone” where the annual cumulative dose was more than 20 mSv and the “Evacuation Zone”, which is the 20 km zone around the 1F nuclear power plants) were chosen for clean-up by the Japanese government. Also, a number of municipalities in eight prefectures in the ICSA, namely Chiba, Gunma, Ibaraki, Iwate, Miyagi, Saitama and Tochigi Prefectures in addition to Fukushima Prefecture, were chosen for conducting surveys in order to determine if clean-up was also necessary in these areas [9]. Details on the municipalities which conducted clean-up based on surveys performed have been published by the MOE [20]. The SDA and ICSA are shown in Figure 1a, b, respectively.

The Japanese government announced in October 2011 that they would aim to reduce additional annual doses (over and above natural background radiation) to 1 mSv or less as a long-term goal [7]. As a consequence of this decision, the areas for clean-up were vast and the Japanese government subsequently had to manage huge volumes of soil and waste generated during the remediation activities [22].

Although at the time (October 2011) there were arguments over the decision that a lower reference level should be selected from the additional dose range of 1–20 mSv/year (and the long-term goal for residents of an additional dose of no more than 1 mSv/year), it was not changed and consequently it took the Japanese government until the 1^st^ of April 2017 to complete all the planned clean-up [21]. By March 2018, the total volume of removed contaminated soil generated by off-site clean-up inside and outside of Fukushima Prefecture reached 17,000,000 m^3^, and over 2.9 trillion Japanese Yen had been allocated as the budget [22].

The next big problem faced by the Japanese Government was attempting to reduce the volume of removed soil and other wastes that would go for disposal. Thus, methods on how to recycle waste materials and where to use these recycled materials should have been considered carefully. According to the MOE, 14, 000, 000 m^3^ of removed soil and waste, including specified waste (>100,000 Bq/kg), is to be stored for a maximum of 30 years [22] at the Interim Storage Facility (ISF) built in Okuma Town and Futaba Town of Fukushima Prefecture. After storage at the ISF, it is intended that waste will be removed from the facility and taken to an as yet unspecified final disposal facility, outside Fukushima Prefecture [23]. It was not easy to acquire land to construct the ISF (it took many years to obtain permission from landowners) and, to date, the strategy for final disposal of this waste has not yet been defined.

Based on scientific justification (e.g. Waddington *et al.* [24]), leaders of the Japanese government who were responsible for environmental remediation should have defined the level of clean-up. Hence, we directed our attention to the decision-making process of the then responsible leaders. Specifically, this study investigates whether or not technically based assessment of dose targets, health effects and risk-based approaches were communicated to the Ministry of the Environment (MOE) which was responsible for the decision-making before the remediation strategy was decided upon by the Japanese government. Further goals are to clarify the roles of academic leaders and define how such decisions were made based on some leadership theories and findings so that future leaders can avoid the same pitfalls encountered during similar events.

## 2 Methodology

The assessment is based on key papers, reports and records of environmental remediation in Fukushima and related issues, in addition to the Role Theory of Kahn *et al.* [25], the Cognitive Resources Theory of Fiedler [26] and the findings of Berkowitz [27] and Bass [28]. In the field of leadership, there are many findings from different approaches such as the trait approach, power-influence approach, behavioral approach, and situational approach. Due to the globally unprecedented disaster and the Fukushima Daiichi accident, the Japanese government was placed in a critical situation and the behavior, including decision-making of the then Minister of MOE, must have been strongly influenced by the situation. Hence, this study focused on the situational approach and used some findings by leadership researchers in addition to situational theories in order to define how the decision to establish the 1 mSv/year dose target was made. According to Yukl [29], “the situational approach emphasizes the importance of contextual factors such as the nature of the work performed by the leader's unit and the nature of the external environment.”

Our assessment is complemented by interviews (between 26^th^ of June 2017 and 7^th^ of November 2017) with a number of experts from both governmental and research organizations:

1. The Director General (technical leader) of the Japan Atomic Energy Agency (JAEA) who was responsible for coordinating environmental remediation in Fukushima prefecture and providing technical support to the MOE (the implementing body)
2. A number of technical experts within JAEA
3. The official of the Fukushima Prefectural Government who was in charge of the environmental remediation
4. Dr. Irena Mele, Head of the Waste Technology Section in the Division of Nuclear Fuel Cycle and Waste Technology of the Department of Nuclear Energy of the International Atomic Energy Agency (IAEA) in 2011
5. International technical experts who were involved in various Fukushima remediation projects
6. Mr. Tomohiro Kondo, the Councilor of the Environmental Regeneration and Materials Cycle Bureau of the MOE
7. Mr. Goshi Hosono, Minister of the MOE between September 2011 and October 2012
8. Mr. Takashi Ohmura, Chief of the Secretariat of the Task Force for Decontamination of the MOE (this job title was translated by Takeuchi because no English title existed just after the accident) since June 2011 and Director, Fukushima Office for Environmental Restoration of the MOE between April 2012 and June 2013.

## 3 Results of the investigation

### 3.1 The Japanese government's decision

According to the interview with the then Minister and officials of the MOE on 10^th^ November 2017 and the additional interview with the then Chief of Secretariat of the Task Force for Decontamination of the MOE on 14^th^ November 2017, the governor and mayors of the municipalities in Fukushima Prefecture demanded an exhaustive clean-up just after the 1F accident and the Japanese government responded to their demand even before the “Act on Special Measures concerning the Handling of Environmental Pollution by Radioactive Materials Discharged by the Nuclear Power Station Accident Associated with the Tohoku District” came into force [30]. This act was intended to clarify the responsibilities of national and local governments, the nuclear power producers and citizens in handling the environmental pollution by radioactive materials discharged during the accident, as well as to promptly reduce the impacts of the pollution from radioactive fallout on human health and the living environment by instituting the measures that should be taken by the national and local governments and the relevant nuclear power producers, etc. [30]; the act came into force from 1^st^ of January 2012.

The Nuclear Safety Commission (NSC) stated the basic view that a lower reference level should be selected from the additional dose range of 1–20 mSv/year and the long-term goal for residents of an additional dose of no more than 1 mSv/year should be achieved based on ICRP Publication 103 [3] of 19^th^ of July 2011 [4]. The average natural radiation background of Japan is 2.1 mSv/year and the average natural radiation background worldwide is 2.4 mSv/year [31]. If the medical exposure in Japan is included, the average dose that the Japanese population receives is around 6 mSv/year [31]. If the long-term goal for residents of an additional dose of no more than 1 mSv/year is set, the exposure dose/year should be reduced to less than the natural radiation background of each area +1 mSv/year after clean-up.

On 26^th^ August 2011, the Nuclear Emergency Response Headquarters decided the fundamental policy on urgent implementation of environmental remediation based on ICRP Publication 103 and the basic view stated by the NSC [5]. Since the then Minister and officials of the MOE did not join the discussions held by the NSC and the Nuclear Emergency Response Headquarters, they did not know details of the discussions when the fundamental policy was being decided. The Minister and officials of the MOE only joined meetings held by the Environmental Remediation Investigation Committee at a later stage, when environmental remediation was carefully discussed [32, 33]. However, the decision that a lower reference level should be selected from the additional dose range of 1–20 mSv/year and the long-term goal for residents of an additional dose of no more than 1 mSv/year was not changed. When we asked during the interview about how to handle the large volumes of contaminated soil, vegetation and other generated wastes, as well as the huge cost of managing the collected radioactive wastes, and whether or not international experts had suggested any different dose targets or strategies, the Minister of the MOE answered, “I visited Fukushima many times and was considerate of the feelings of mothers who had young children and understood their fears of radiation because I also had an elementary school child. The dose target issue was discussed in depth by excellent technical experts such as university professors. I also met international experts from the IAEA and so on. All of them supported our decision. If the same accident occurs again, I will set the final dose target of no more than 1 mSv/year again. I'm sure any country would set the same target as ours if a similar accident occurs.”

### 3.2 Recommendation of international and Japanese experts

All the international and Japanese experts who were interviewed suggested that the dose target of 1 mSv/year was too low and a dose of 5 mSv/year would have been more reasonable, even taking into account young children. All the technical experts interviewed were also worried about the management of the much larger volumes of generated waste which resulted from the lower dose target. They recommended that the budget allocated for remediation should have been spent for some other purpose. Ahn [34] also questioned “How clean is clean enough?” and argued about the total volume of waste material, the associated cost and the insignificant health risks in areas of low contamination were factors that should have been considered.

According to Kurokawa's report [1], some residents wanted to remain in their homes and actively support clean-up, but others wanted to move away and requested compensation to support their relocation. Many people thought it would be impossible to resume their normal lives and hoped the government would spend the allocated budget to support evacuees in starting new lives outside Fukushima Prefecture. Some also hoped that the Japanese government would re-examine the dose target of 1 mSv/year [35, 36]. When members of the IAEA visited Fukushima in 2011, they also suggested not to be overly sensitive to safety and pointed out that the dose target of 1 mSv/year was inappropriate. The Minister of the MOE also recognized the IAEA's advice, but he answered at the press conference held on 18^th^ October 2011 that he would follow the wishes of municipalities in Fukushima Prefecture [37]. On 4th March 2013, he objected to the article published in Yomiuri Shimbun (a Japanese newspaper) that his decision of 1 mSv/year hindered evacuees' return to their homes [38]. He explained in his blog that, although he pointed out repeatedly that 1 mSv/year was not the standard for health or evacuees' return, the target of 1 mSv/year was set according to the demands of the mayors and the governor of Fukushima Prefecture [39].

Fears were also generated by the media. Exaggerated news reports provoked suspicion and resentment not only towards TEPCO and the Japanese government but also scientific experts in nuclear technology from any of the involved organizations. According to interviews with JAEA staff and international experts, they had a strong impression that public opinion was created and controlled by the media. The interviewees from JAEA said mothers who had young children were frightened of the radiation and cried a lot and emphasized that the media created an atmosphere of fear. As the then Minister of the MOE said, those frightened mothers, in addition to the governor and the mayors of the municipalities in Fukushima Prefecture, played a major role in leading to his decision of implementing exhaustive clean-up and the subsequent setting of the long-term dose goal of no more than 1 mSv/year for the residents. Although the Minister of the MOE insisted that all technical experts supported the Japanese government's decision, we could not find any technical experts who supported the target of 1 mSv/year above background.

### 3.3 How was the decision to establish the 1 mSv/year dose target made by the Minister of the MOE?

In this section, we focus on the situational approach and examine the decision-making process based on situational theories. Yukl [29] described one of the situational theories, the Role Theory of Kahn *et al.* [25] as that “the role expectations from superiors, peers, subordinates, and outsiders are major influence on a leader's behavior and leaders adapt their behavior to role requirements, constraints, and demands of the leadership situation.” For the environmental remediation in Fukushima, the role expectations from the governor and mayors of municipalities in Fukushima Prefecture and many frightened mothers with children must have been the major influence on the Minister and officials of the MOE who were responsible for the decision-making. They adapted their behavior to role requirements, constraints, and demands of the leadership situation, namely an exhaustive clean-up without thought of cost, time and environmental impacts.

Yukl [29] explained that situational variables such as interpersonal stress determine whether a leader's intelligence and experience enhance group performance. According to the Cognitive Resources Theory of Fiedler [26], leaders use their intelligence when stress is low, but their experience when stress is high. The experience of a leader is related to group performance under high stress but not under low stress because an experienced leader most likely relies mainly on experience to solve problems when under high stress, not on intelligence [29]. When the then Minister and officials of the MOE had to set the long-term additional dose target for residents, stress was very high, which likely interfered with the use of intelligence (rationality) to solve problems and make decisions. Needless to say, these leaders did not have any prior experience in the clean-up of radioactive materials.

Bass [28] explained that “the leadership that succeeds in influencing followers may not be most effective in stressful situations, particularly in the long run”. The leadership by the then Minister of the MOE succeeded in influencing people, but it resulted in a “faulty decision made too hastily” [28] and “a defensive reaction” [28] to set the target of 1 mSv/year, even if his leadership was likely to “contribute to escape from panic situations” [28], in particular for frightened mothers with young children. His leadership decision on the exhaustive clean-up must have eased the concerns of such mothers and the mayors of the municipalities in Fukushima at least in the interim.

**Figure 2:**
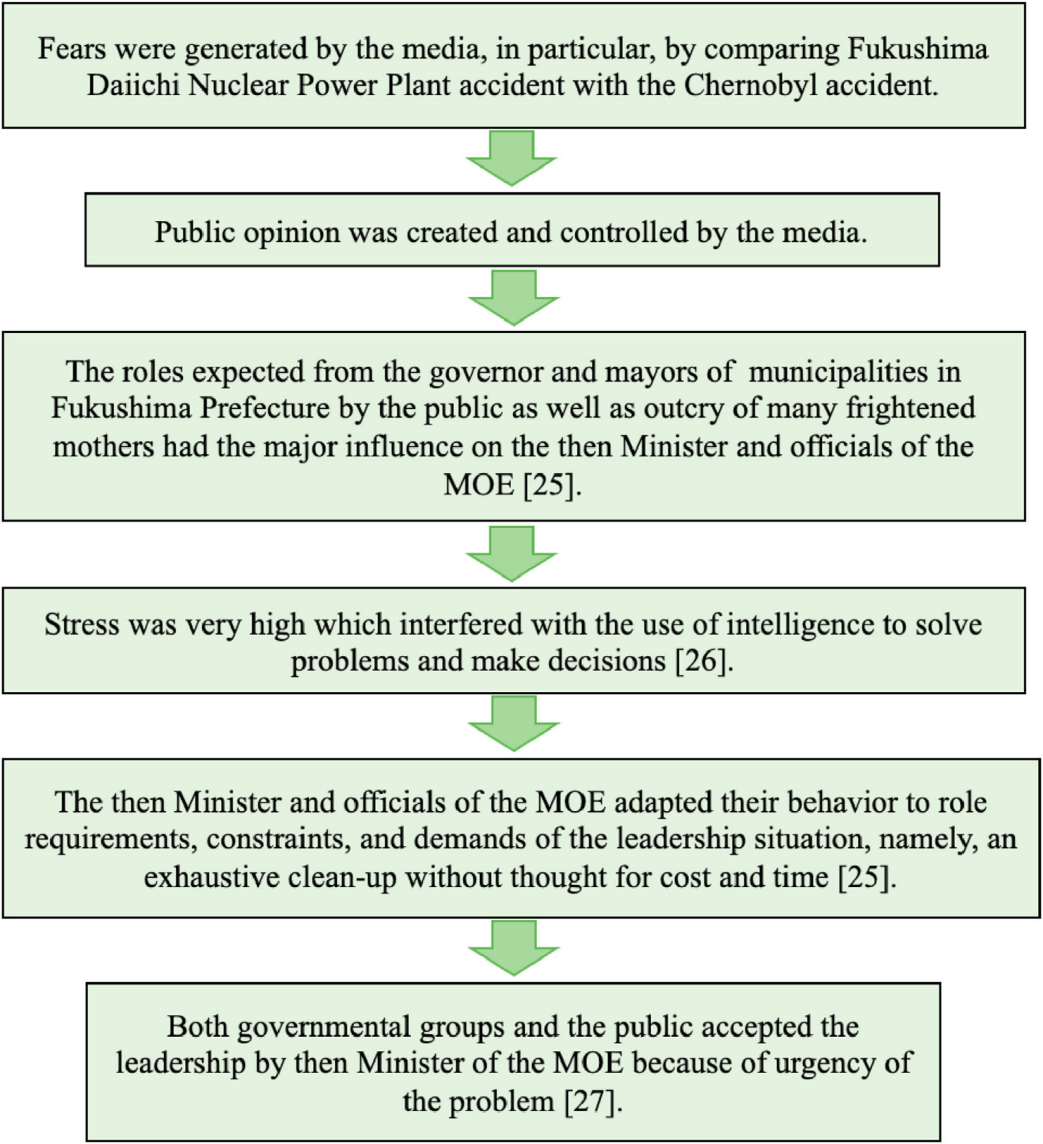
Diagram showing how the decision of establishing the dose target of 1 mSv/year was made by the then Minister of the Ministry of the Environment (MOE) and accepted by governmental groups and the Japanese public.

Berkowitz [27] pointed out that when there are urgent problems confronting groups, the group motivation to reach a solution as quickly as possible appears to be stronger than their motivation regarding the expectations concerning role differentiation (expectation that a leader should be functionally differentiated from the others in the group). There is also a tendency for these groups to have greater interdependence among the members [27]. Bass explained that Berkowitz found that “both governmental and industrial groups were more likely to accept leadership when the problem was urgent” [28]. Since the radiation problem was urgent and officials, politicians and the general public, including residents in Fukushima, were under high stress just after the 1F accident, they were likely to accept the leadership of the then Minister of the MOE despite the content of his decision. Understanding the decision-making process as shown in Figure 2 can help future leaders to avoid the same pitfalls encountered under similar events.

## 4 The leadership role of academics

### 4.1 What should have been done by Japanese academic leaders?

Fukushima cannot be rejuvenated by environmental remediation alone. Although it is important, political and administrative leaders should also create a clear vison and strategy for rejuvenation at the earliest stages, so that they can allocate their limited finances and human resources appropriately towards an integrated plan for reconstruction, which includes environmental remediation. For example, it was necessary to sustain local infrastructure such as roads and water supply facilities. Although finances were allocated for that purpose by both the Japanese Government and the local government (Fukushima Prefecture), they were insufficient, and according to the interview with the officials of Fukushima Prefecture, the infrastructure in the Prefecture requires further improvement. The Japanese government became too sensitive to the feelings of the residents of Fukushima and focused only on environmental remediation immediately after the accident. It took too much time to complete environmental remediation due to the dose target of no more than 1 mSv/year being set.

Nine years have passed since the 1F accident but only 28% of the registered residents of the municipalities where evacuation orders have been lifted have returned to their homes according to a 2020 survey [40, 41]. About 70% of the residents who were evacuated from Fukushima municipalities settled in different municipalities outside Fukushima Prefecture. For the right decision-making on environmental remediation, a technically based assessment of dose targets, health effects and risk-based approaches should have been logically communicated by Japanese scientific leaders (usually most scientific leaders are academic leaders in Japan) before the dose target was set, not only to the then Minister and officials of the MOE but also to the governor and the mayors of the municipalities in Fukushima Prefecture.

The Minister and officials of the MOE should have consulted international technical experts who had experience or knowledge in environmental remediation at the earliest stage, not after the decision was made. International technical experts can suggest reasonable dose targets, but ultimately, they cannot object to a target decided by the Japanese government. Such experts should have been a part of a team to help explain what would have been realistically achievable and why. Furthermore, it would have been desirable for Japanese academic leaders to provide the appropriate information on environmental remediation and reconstruction for Fukushima to the then Minister and officials of the MOE. In collaboration with Japanese academic leaders and international technical experts, the Minister and officials of the MOE could also have developed a communication program to alleviate residents' fears and expedite reconstruction for the affected Fukushima municipalities.

### 4.2 The Windscale accident

By examining experience in recovery from nuclear accidents that took place in the past, academic leaders could have provided information that was directly relevant for the Fukushima case. Based on this case, environmental remediation could have been planned and implemented more effectively whilst maintaining the highest possible safety standards and balancing the economic burden (both of which impact the Japanese public). For example, academic leaders could have examined the case of Cumbria in NW England. The region of Cumbria, a popular tourist destination in the United Kingdom, has twice been contaminated with radiocesium released from nuclear reactor accidents; once in 1957 after the Windscale fire [11, 12] and again in 1986 after the catastrophic explosion at the Chernobyl NPP [10]. Research into both of these cases could have informed and helped guide the development of appropriate and practical measures to be implemented after the 1F accident.

**Figure 3:**
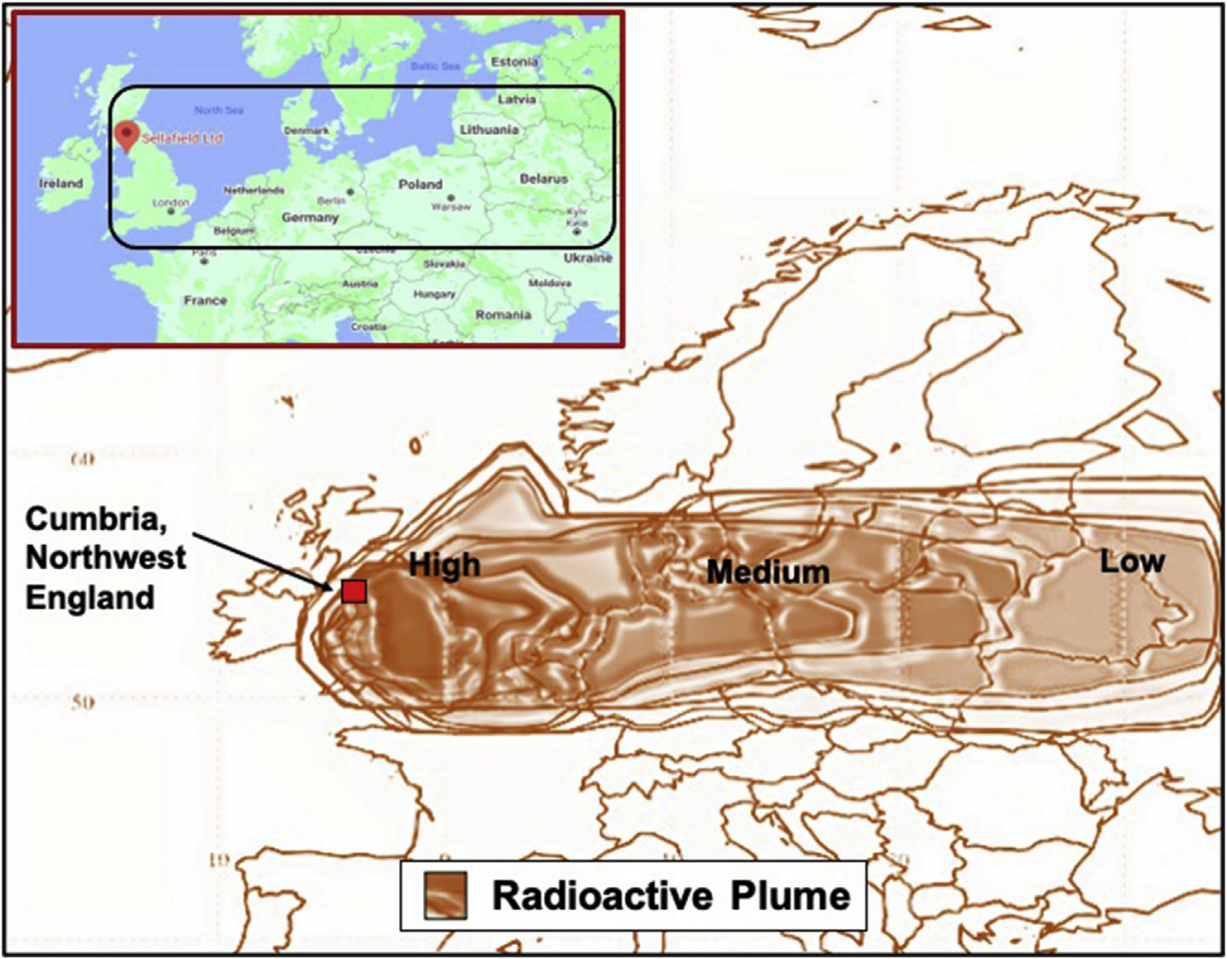
Map showing the spread of the radioactive cloud during the Windscale nuclear reactor fire, Cumbria, England on the 10^th^ of October 1957. Map redrafted from Johnson *et al.* [43].

In the case of Cumbria, no remediation was performed after either accident, although some restrictions were placed on foodstuffs such as milk after the Windscale accident [10, 14, 42]. Similarly, no extensive off-site remediation was performed at Chernobyl; however public access to highly contaminated regions was restricted [10, 42]. Although the Windscale accident was not well known even among technical experts in Japan, it was perhaps the most analogous to Fukushima's case (Figure 3, Table 3). Taking a closer look, the Windscale nuclear reactors were built on the coast of Cumberland (now part of Cumbria), Northwest England to produce plutonium and other nuclear materials for the UK nuclear weapons program between the years 1947 and 1951. The two reactor piles at Windscale used graphite as a neutron moderator which allows a combination of natural and (from late 1953) slightly enriched uranium metal to be used as fuel [11] and for Wigner energy accumulation and release [12]. On the 10th of October 1957, the release of Wigner energy at Windscale Pile Number 1 through a standard annealing operation was not properly controlled. This resulted in the overheating of the core and subsequent burning of fuel and graphite in the air coolant [11]. As the fire took place, radioactive materials such as fission and activation products from a small percentage of the core were released into the atmosphere. This nuclear disaster is the largest recorded release of radioactive material in the history of the nuclear industry in the UK [11]. Despite this, the British government did not conduct any clean-up, even though the radioactive cloud travelled southeast across most of England and then further eastwards over northern and western Europe (Figure 3).

**Table 3:** Inventory of radionuclides released during the Fukushima and Windscale nuclear accidents in 2011 and 1957, respectively.

Nuclear Accidents	Oceanic Release (Bq)				Atmospheric Release (Bq)			
	^134^Cs	^137^ Cs	^131^I	^210^Po	^134^Cs	^137^ Cs	^131^I	^210^Po
Fukushima [44]	3.5 × 10^15^	3.6 × 10^15^	1.1 × 10^16^	-	1 × 10^16^	1 × 10^16^	5 × 10^17^	-
Windscale [11]	-	1.8 × 10^13^^a^	1.8 × 10^14^^a^	4.2 × 10^12^^a^	-	1.6 × 10^14^^b^	1.6 × 10^15^^b^	3.8 × 10^13^^b^

a Taking into account that oceanic release from Windscale was limited by 10% of total radioactive material.

b Taking into account that atmospheric release from Windscale was limited by 90% of total radioactive material.

Nonetheless, Cumbria is now one of the most popular places for sightseeing in the UK. Fukushima used to be known as a beautiful place for sightseeing before the accident but has suffered reputational damage, in stark contrast to Cumbria. If political and administrative leaders and residents of Fukushima had learned lessons from the Cumbrian case, they could have devised a more effective and less damaging program for the reconstruction of Fukushima, without undertaking unnecessary clean-up that resulted in significant quantities of radioactive waste having to be managed.

### 4.3 Leadership and COVID-19

The world is now facing a pandemic, in the absence of any kind of guidelines on which to base national and international responses. To counter COVID-19, political leaders from different countries have been choosing different interactions or measures. At the beginning of 2020, the concerns of political leaders and people in most countries, except Sweden, were only how to minimize the number of confirmed cases and deaths, similar to the concerns of the Japanese political leaders and people after the 1F accident.

The Swedish government successfully implemented their controversial COVID-19 strategy without inciting strong public opposition. By looking closely at the situation in Sweden and comparing it to what happened during the 1F accident, the same strategy could be explored by the Japanese government for future consideration. The strategy of the Swedish government in facing COVID-19 was more relaxed compared to other western countries. The government chose to adopt COVID-19 safety measures whilst also minimizing the impact on their domestic economy [45], thus making them the only European country which implemented a more sustainable strategy.

Although the number of deaths in Sweden is higher than the other countries in Fenno-Scandinavia (as of 29^th^ of October 2020, Sweden: 5929, Denmark: 716, Finland: 354, Norway: 281) [46], more than 80% of Sweden's residents think their country's approach was the right one [47]. In Sweden, the Public Health Agency, an independent organization of experts, is responsible for public health issues. The government and the parliament of Sweden respect the autonomy of the Public Health Agency and their strategies in response to COVID-19, which were planned and recommended by the Public Health Agency, can be implemented smoothly. Sweden's residents trust their government and/or the Public Health Agency because of their transparency, resulting in high public acceptance of the COVID-19 strategies recommended by the Public Health Agency.

If, during the 1F accident, there had been such a reliable organization which consisted of Japanese experts, perhaps these experts could have recommended a much better clean-up strategy to the then Minister of the MOE, and, as a result, Japan would not have wasted so much time and money on inappropriate clean-up. Although the MOE was the responsible organization for the clean-up in Japan, they did not have any prior experience or knowledge in this area and relied on an expert (usually most experts are academics in Japan) committee for advice.

To complicate matters, Japan has a peculiar organizational culture in which political and/or administrative and/or academic leaders do not clarify who is responsible for each decision and most of the important decisions are made without open debate. Hence, after the 1F accident, the pros and cons of setting the dose target of no more than 1 mSv/year was not communicated clearly to the general public. With regard to the current global pandemic, the Japanese government did not disclose most of the minutes of the COVID-19 expert meeting (established on 14^th^ February and abolished on 3^rd^ July 2020) [48] where the national response was discussed, and there is criticism here as well concerning the secretive nature [49].

Leadership for reconstruction/rejuvenation of the affected areas of Fukushima Prefecture, including clean-up, cannot be practical or effective without sound scientific justification. Appropriate scientific input should have been provided by academic leaders to the responsible political and administrative leaders and such scientific justification should have been disclosed (in an easily understandable manner) to the general public, including the residents of Fukushima Prefecture, so that the general public could develop trust in their leaders and more readily understand and accept their decisions. The leadership role of academics in Japan needs to be examined radically in the future. Furthermore, political, administrative and academic leaders are the main actors who can change the peculiar organizational culture in Japan. It is high time for such leaders to reconsider and change this situation in order to make better decisions and to create a better nation in the future.

## 5 Conclusions

Technically based assessments of dose targets, health effects and risk-based approaches of experts who had experience or the necessary knowledge were not communicated to the then Minister and officials of the MOE before the remediation strategy was decided upon. This is the main reason why the Minister of the MOE announced the long-term goal for residents of reducing the additional radiation dose to no more than 1 mSv/year.

The expectations from the Governor and the Mayors of the municipalities in Fukushima Prefecture, and from many frightened people, in particular mothers with young children, were the major influence on the then Minister and officials of the MOE. When they set the long-term dose target, stress levels were very high and interfered with the use of intelligence (rationality) to solve problems and make decisions. They adapted their behavior to role requirements, constraints, and demands of the leadership situation, namely an exhaustive clean-up without due consideration of the resulting environmental impacts, costs and time required. Since the radiation problem was urgent and officials, politicians and the general public, including residents in Fukushima Prefecture, were under high stress just after the 1F accident, they were likely to accept the leadership of the Minister of the MOE.

Academic leaders could have examined the Windscale accident which can be considered to be much more analogous to the 1F accident than the accident that took place at Chernobyl. Environmental remediation could have been planned and implemented more effectively, while still maintaining the highest possible safety standards and balancing the economic burden, both of which impact the Japanese public. Appropriate scientific input should have been provided based on this type of experience and presented to the political and administrative leaders. In addition, such scientific justification should have been presented (in an easily understandable manner) to the general public, including the residents of Fukushima Prefecture, so that the general public could have developed more trust in their leaders and more readily accept their decisions.

How to change the peculiar working culture of Japan, in which political and/or administrative and/or academic leaders do not clarify who is responsible for each decision and most of the important decisions that are made without open debate, should be an important research theme for the future. As we are currently facing a pandemic, now is a key opportunity to discuss leadership roles of academics and how to change the culture.

## Declarations

### Author contribution statement

Maria R. H. Takeuchi: Conceived and designed the experiments; Performed the experiments; Analyzed and interpreted the data; Contributed reagents, materials, analysis tools or data; Wrote the paper.

Tatsuya Hasegawa: Conceived and designed the experiments.

Susie M. L. Hardie: Contributed reagents, materials, analysis tools or data; Wrote the paper.

Linda E. McKinley: Analyzed and interpreted the data; Wrote the paper.

Gian Powell Marquez: Analyzed and interpreted the data; Contributed reagents, materials, analysis tools or data; Wrote the paper.

Keiichi N. Ishihara: Conceived and designed the experiments; Contributed reagents, materials, analysis tools or data.

### Funding statement

This research did not receive any specific grant from funding agencies in the public, commercial, or not-for-profit sectors.

### Data availability statement

Data will be made available on request.

### Declaration of interests statement

The authors declare no conflict of interest.

### Additional information

No additional information is available for this paper.
